# DNA methylation in schizophrenia in different patient-derived cell types

**DOI:** 10.1038/s41537-016-0006-0

**Published:** 2017-01-23

**Authors:** Alejandra M. Vitale, Nicholas A. Matigian, Alexandre S. Cristino, Katia Nones, Sugandha Ravishankar, Bernadette Bellette, Yongjun Fan, Stephen A. Wood, Ernst Wolvetang, Alan Mackay-Sim

**Affiliations:** 1 Griffith Institute for Drug Discovery, Griffith University, Nathan, QLD Australia; 2 Instituto de Biologia y Medicina Experimental-IBYME-CONICET, Buenos Aires, Argentina; 3 The University of Queensland Diamantina Institute, Translational Research Institute, Brisbane, QLD Australia; 4 Queensland Centre for Medical Genomics, Institute for Molecular Bioscience, The University of Queensland, St Lucia, Brisbane, QLD Australia; 5 Australian Institute for Bioengineering and Nanotechnology, The University of Queensland, Brisbane, QLD Australia

## Abstract

DNA methylation of gene promoter regions represses transcription and is a mechanism via which environmental risk factors could affect cells during development in individuals at risk for schizophrenia. We investigated DNA methylation in patient-derived cells that might shed light on early development in schizophrenia. Induced pluripotent stem cells may reflect a “ground state” upon which developmental and environmental influences would be minimal. Olfactory neurosphere-derived cells are an adult-derived neuro-ectodermal stem cell modified by developmental and environmental influences. Fibroblasts provide a non-neural control for life-long developmental and environmental influences. Genome-wide profiling of DNA methylation and gene expression was done in these three cell types from the same individuals. All cell types had distinct, statistically significant schizophrenia-associated differences in DNA methylation and linked gene expression, with Gene Ontology analysis showing that the differentially affected genes clustered in networks associated with cell growth, proliferation, and movement, functions known to be affected in schizophrenia patient-derived cells. Only five gene loci were differentially methylated in all three cell types. Understanding the role of epigenetics in cell function in the brain in schizophrenia is likely to be complicated by similar cell type differences in intrinsic and environmentally induced epigenetic regulation.

## Epigenetics: A genome-wide picture in patient-derived cells

Schizophrenia-associated differences in the DNA methylation status of patient-derived cells suggest it could affect early brain development. Mechanisms that control gene expression without altering the genetic code, such as DNA methylation, could explain how environmental risk factors contribute to schizophrenia in genetically susceptible individuals. Alan Mackay-Sim and colleagues from Griffith University, Australia, carried out genome-wide comparisons of DNA methylation in induced pluripotent stem (iPS) cells, olfactory neurosphere-derived cells and fibroblasts from patients and controls. Differences in the DNA methylation pattern between patient and control iPS cells, which could reflect what happens in the embryo, suggest a disease-associated effect very early on in development. Only five genes were differentially methylated in all three patient-derived cell types compared to controls. None of these genes has previously been associated with schizophrenia and may represent new targets for future research.

## Introduction

Schizophrenia is recognized as a polygenic disorder with the contribution of potentially hundreds of risk genes that affect brain development.^1^ Environmental risk factors acting during early development and into young adulthood also contribute to schizophrenia in susceptible individuals. From a neurobiological perspective, environmental factors must act ultimately on cells in the nervous system to change the way they act, or interact, in the neuronal networks that determine behavior. This may occur through epigenetic mechanisms that alter gene expression without affecting the genetic code via modifications of DNA and DNA-associated histone proteins by acetylation, phosphorylation, and methylation.^2, 3^ Even the social environment can act epigenetically: maternal grooming of rat pups reduced DNA methylation of the glucocorticoid receptor gene promoter in the hippocampus, increasing transcription factor binding, and reducing the hypothalamic-pituitary-adrenal stress response in adulthood.^4^ Such observations have helped shape the view that epigenetics is a potential “non-genetic” factor leading to both causes and effects in neuropsychiatric disorders.^5, 6^ Thus, the “biological” environment during development in utero or following birth, such as prenatal infections^7^ and vitamin D status,^8^ as well as the “social” environment, such as migrant status^9^ and childhood trauma,^10^ might act on the brain via epigenetic mechanisms to alter gene expression, brain development, and ultimately behavior, leading to schizophrenia in genetically susceptible individuals.^5, 6, 11^

The majority of studies of epigenetic modifications in schizophrenia are DNA methylation analyses targeted to specific genomic regions of candidate genes (reviewed in ref. 11), but recent developments in technology have allowed broader, genome-wide comparisons of DNA methylation in schizophrenia patients and unaffected controls in postmortem brain^12, 13^ and in leukocytes.^14^ One aim of this study was to determine whether there is any schizophrenia-associated DNA methylation in patient-derived induced pluripotent stem (iPS) cells that could indicate the influence of genetic risk factors very early in development. Olfactory neurosphere-derived (ONS) cells and fibroblasts provide contrast between schizophrenia-associated DNA methylation in adult cells from neural and non-neural origins. A second aim was to determine whether schizophrenia involves DNA methylation that is carried into adulthood, exemplified by patient-derived ONS cells and fibroblasts. DNA methylation regulates gene expression, so it was also of interest to explore mRNA expression profiles in the three cell types. The final aim of this study was to identify which cell functions would be affected by schizophrenia-associated differences in DNA methylation and gene expression. These aims were achieved by obtaining genome-wide DNA methylation and gene expression profiles from iPS cells, ONS cells, and fibroblasts from the same patients, and controls were obtained and the schizophrenia-associated genes were subjected to functional annotation and pathway analysis to identify affected cell functions and processes.

## Results

### DNA methylation and gene expression defined the three cell types

**Fig. 1:**
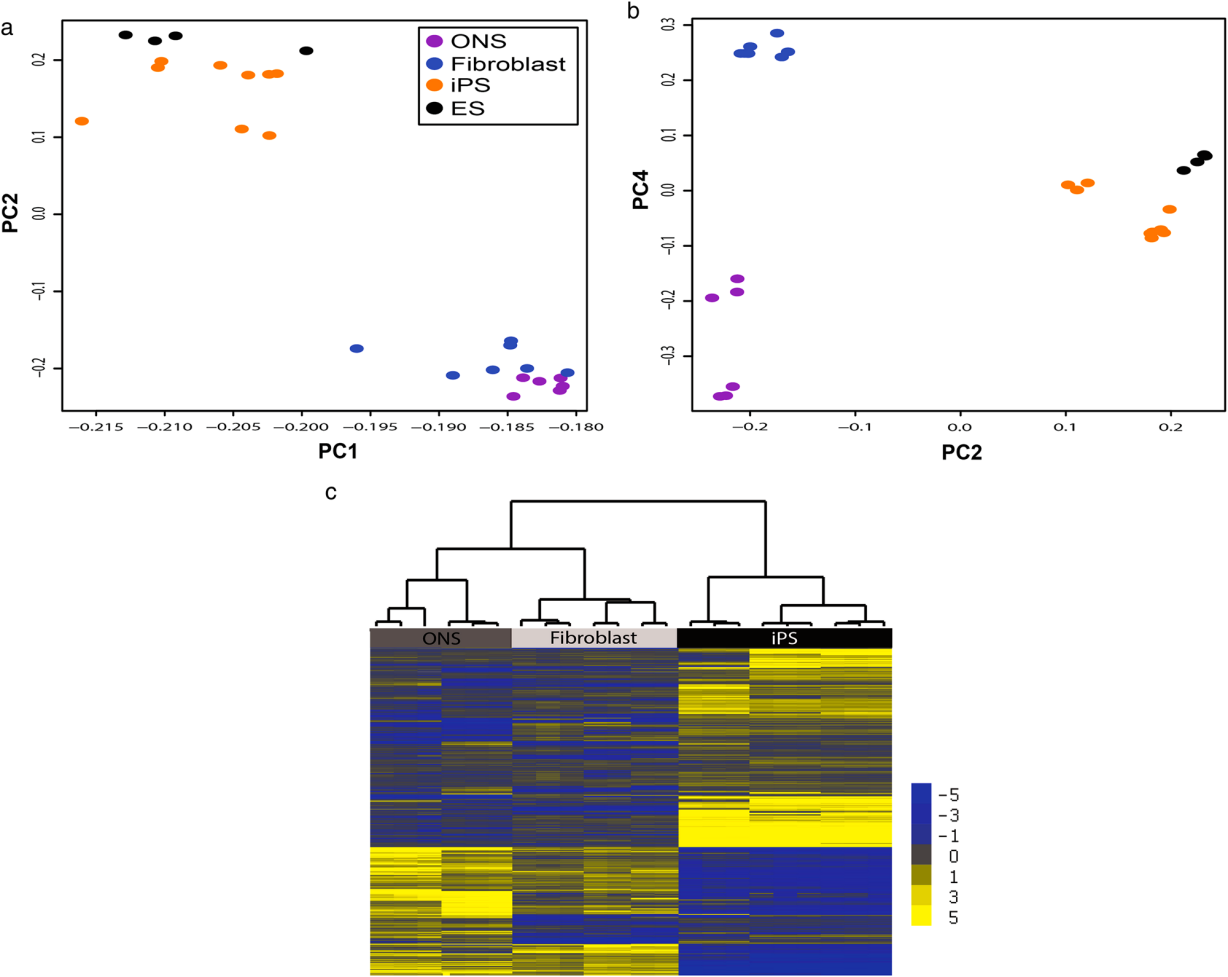
Cell types have distinct DNA methylation profiles. **a**, **b** Unbiased PCA on all detected probes on array demonstrates that cell types are distinguished by DNA methylated gene loci. **a** Principal components 1 (*x-axis*) and 2 (*y-axis*) separate the pluripotent cells (iPS and ES cells) from the adult tissue-derived cells (ONS and fibroblasts). **b** Principal components 2 (*x-axis*) and 4 (*y-axis*) separate the three cell types from each other. **c** The individual cell lines separate into cell types after cluster analysis on the probes that were statistically different between the cell types (*p* < 0.05, FDR correction). Gene loci are illustrated as *horizontal yellow* and *blue lines* scaled from the average, which is *gray*. Higher methylation than average is *yellow*. Lower than average is *blue*. Scale is on right. Statistical clustering similarity is shown by the tree diagram. iPS cells are distinctly different from the adult-derived cells (top branch). ONS and fibroblasts are also distinguished from each other (second branch on left)

Global methylation status of the three cell types was compared by principal components analysis using the *M*-value of every gene/probe on the array without any statistical filter applied. Principal components 1 and 2 distinguished the pluripotent cells, iPS cells, and embryonic stem cells (ES cells), from the non-pluripotent cell types (ONS cells and fibroblasts; Fig. 1a). Principal components 2 and 4 distinguished the ONS cells from the fibroblasts and each from the pluripotent cells (Fig. 1b). The individual probes that defined the differences between the cell types were identified by comparing, in a pairwise manner, the *M*-value of the CpG loci of iPS cells, ONS cells, and fibroblasts, applying a robust statistical threshold [*p* < 0.0001, with Benjamini and Hochberg False Discovery Rate (FDR) Correction for multiple testing]. There were 7,854 CpG loci that were differentially methylated among the three cell types, 28% of the total detected probes on the array, while the majority of detected gene loci (69%) were methylated similarly in all cells, independently of cell type or disease status. The significant CpG loci were used as input to an unsupervised hierarchical cluster analysis, which identified three main clusters that distinguished between the different cell types (Fig. 1c). The cluster analysis separated the individual cell lines into three cell types, as shown by the tree structure at the top of the analysis in Fig. 1c.

**Fig. 2:**
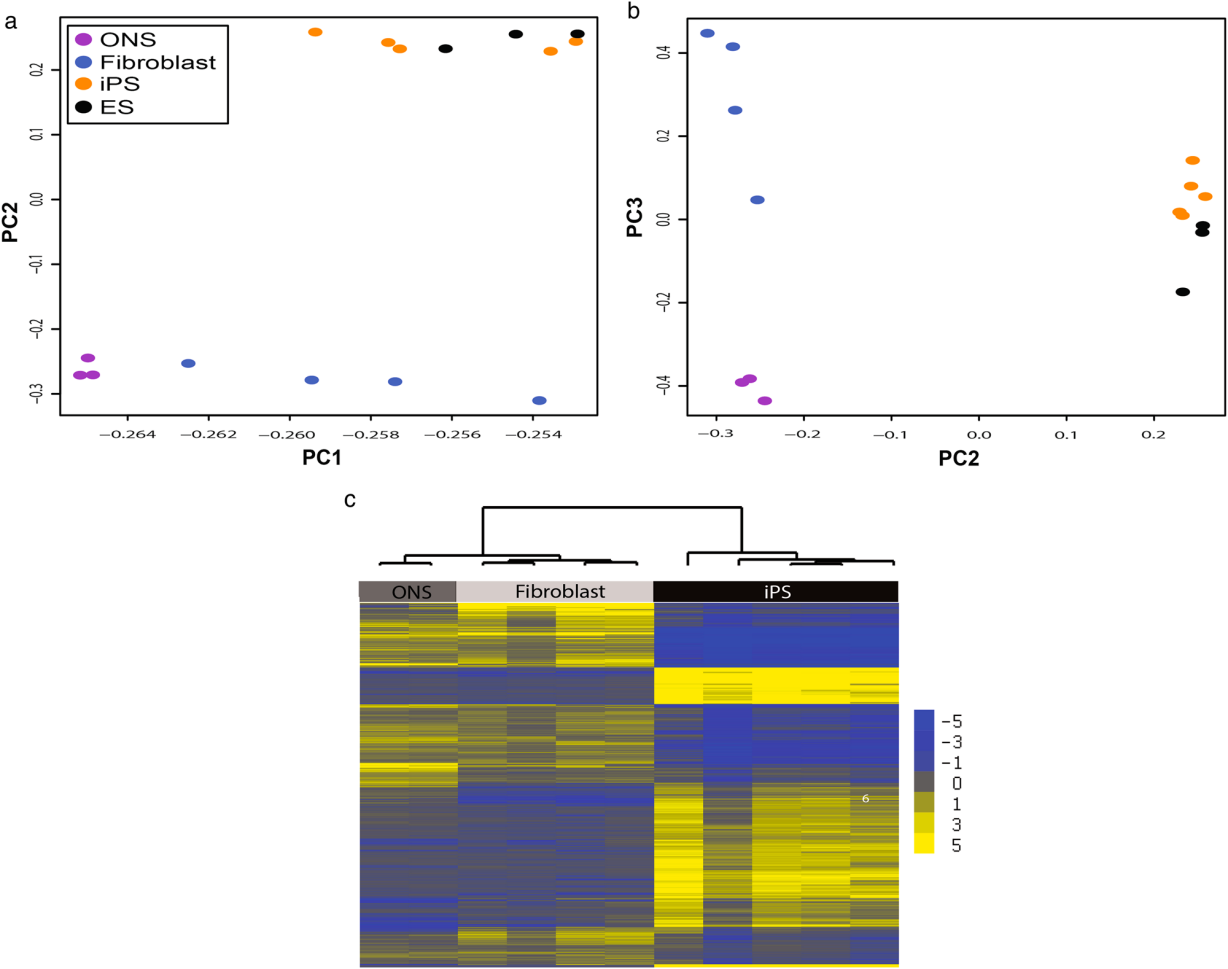
Cell types have distinct gene expression profiles. **a**, **b** Unbiased PCA on all detected probes on array demonstrates that cell types are distinguished by the genes they express. **a** Principal components 1 (*x-axis*) and 2 (*y-axis*) separate the pluripotent cells (iPS and ES cells) from the adult tissue-derived cells (ONS and fibroblasts) and also separate the ONS and fibroblasts. **b** Principal components 2 (*x-axis*) and 3 (*y-axis*) separate the three cell types from each other. **c** The individual cell lines separate into cell types after cluster analysis on the probes that were statistically different between the cell types (*p* < 0.05, FDR correction). Genes are illustrated as *horizontal yellow* and *blue lines* scaled from the average, which is *gray*. Higher expression than average is *yellow*. Lower than average is *blue*. Scale is on right. Statistical clustering similarity is shown by the tree diagram. iPS cells are distinctly different from the adult-derived cells (top branch). ONS and fibroblasts are also distinguished from each other (second branch on the left)

Global gene expression status of the three cell types was compared by principal components analysis using the raw fluorescence value of every gene/probe on the array after normalization and background correction without any statistical filter applied. Principal components 1 and 2 distinguished the pluripotent (iPS cells and ES cells) from the non-pluripotent cell types (ONS cells and fibroblasts; Fig. 2a). Principal components 2 and 3 distinguished ONS cells from fibroblasts and each from the pluripotent cells (Fig. 2b). The individual genes that defined the differences between the cell types were identified by comparing, in a pairwise manner, the fluorescence value for each gene probe of iPS cells, ONS cells, and fibroblasts, applying a robust statistical threshold (*p* < 0.0001, with Benjamini and Hochberg FDR correction for multiple testing). This analysis revealed 8231 genes/probes that were differentially expressed among the three cell types, 41% of the probes detected on the array (*n* = 20,206). These significant probes were used as input to an unsupervised hierarchical cluster analysis, which identified three main clusters that distinguished between the different cell types (Fig. 1c). The cluster analysis separated the individual cell lines into three cell types, as shown by the tree structure at the top of the analysis in Fig. 2c.

### Schizophrenia-associated DNA methylation in the three cell types

A small number of probes on the array were differentially methylated among cell lines derived from schizophrenia patients and controls. These represented 3–5% of the probes depending on the cell type: in iPS cells there were 883 CpG loci with statistically significant patient-control differences (689 genes, Supplementary Table 1, *p* < 0.05, with Benjamini and Hochberg FDR correction for multiple testing), in ONS cells there were 1328 CpG loci (1221 genes, Supplementary Table 2), and in fibroblasts there were 952 (859 genes, Supplementary Table 3). Five gene loci (0.02% of differentially methylated loci or genes) were methylated differentially in the same way between patients and controls in all three cell types (Supplementary Table 4). Although small, this overlap was not expected by chance (*p* = 0.016, estimated by simulating 10,000 random selections from the three gene lists from the total pool of 27,578 genes). Gene loci that were significantly hypomethylated in all patient cell types compared to control cells were *PSMD5*, *LRRN4/C20orf75*, *FAM20B*, and *AEN/ISG20L1*. One gene locus was significantly hypermethylated in patient cells (*ID2*).

The CpG loci that were differentially methylated in patient-derived and control-derived cells were subject to Gene Ontology (GO) analysis. GO categories and functional annotations within the categories, are shown in Table 1, which shows the top categories ranked on statistical significance (*p*-value). Several GO categories were identified in all cell types: “cellular growth and proliferation”, “cell death and survival”, “carbohydrate metabolism” and “cellular movement”. Although the defined GO categories were similar among the cell types, the schizophrenia-associated differences are composed of different individual genes that assemble into different functions within each GO category (Table 1). Pathway analysis of the differentially methylated loci identified one pathway common to all the three cell types (“LPS/IL-1 mediated inhibition of RXR function”) and 14 shared by ONS cells and fibroblasts (Supplementary Table 5, showing the pathways passing statistical significance at *P* < 0.05).

**Table 1:** GO categories of genes differentially methylated between patients and controls (top categories ranked according to *p*-value)

Category	Functional annotation	*p*-value	# Molecules
iPS cells			
Cellular growth and proliferation	Proliferation of tumor cell lines	0.00835	50
	Proliferation of cells	0.02000	81
Cellular function and maintenance	Autophagy of cells	0.00049	10
	Autophagy	0.00107	13
Carbohydrate metabolism	Synthesis of carbohydrate	0.00402	23
	Quantity of carbohydrate	0.00544	25
Cell cycle	G2 phase of tumor cell lines	0.00144	8
	Arrest in G2 phase of tumor cell lines	0.00202	7
Cell death and survival	Cell death of breast cancer cell lines	0.0109	16
	Apoptosis of breast cancer cell lines	0.0312	13
Cellular movement	Cell movement	0.021	55
ONS cells			
Cellular movement	Cell movement	2.41E-16	254
	Migration of cells	7.03E-12	205
Cellular growth and proliferation	Proliferation of cells	1.79E-14	379
	Proliferation of tumor cell lines	7.98E-09	210
Carbohydrate metabolism	Metabolism of carbohydrate	6.88E-13	145
	Quantity of carbohydrate	3.12E-09	104
Cell death and survival	Cell death	4.34E-15	416
	Necrosis	3.27E-13	362
Lipid metabolism	Synthesis of lipid	1.09E-09	144
	Fatty acid metabolism	1.52E-07	112
Fibroblasts			
Cellular growth and proliferation	Proliferation of cells	5.03E-10	408
	Proliferation of liver cells	4.59E-04	48
Cellular movement	Cell movement	4.86E-10	264
	Migration of cells	3.54E-07	215
Lipid metabolism	Concentration of lipid	5.93E-09	186
	Synthesis of lipid	5.16E-08	161
Carbohydrate metabolism	Quantity of carbohydrate	8.01E-05	105
	Metabolism of carbohydrate	1.67E-04	134
Cell death and survival	Cell death	1.76E-12	459
	Necrosis	1.62E-09	393

### Schizophrenia-associated gene expression in the three cell types

Differentially expressed genes in the iPS cells from patients and controls were defined as those with statistically significant fluorescence values after normalization and background correction (*p* < 0.05, with Benjamini and Hochberg FDR correction for multiple testing). Differentially expressed genes in the ONS cells and fibroblasts were identified by reanalysis of our previous data^15^ using the same statistical criteria as used for iPS cells. The differentially expressed genes in iPS, ONS cells and fibroblasts were then subject to GO analysis. In iPS cells there were four GO categories made up of 13 functional annotations that were significantly different between patient-derived and control-derived iPS cells (Table 2). In ONS cells there were six categories and eleven functional annotations that were significantly different between patient and control-derived iPS cells (Table 2). Fibroblasts had few differences in gene expression and no significant GO categories. Most of the differences between patient and control iPS and ONS cells involve basic cell functions and both shared the category “Cell Movement” (Table 2).

**Table 2:** GO categories of genes differentially expressed between patients and controls

Category	Functional annotation	*p*-Value	Predicted activation state	Activation *Z*-score	# Molecules
iPS cells					
Cellular assembly and organization	Organization of cytoskeleton	4.38E-05	Increased	2.1	129
	Microtubule dynamics	4.13E-04	Increased	2.3	101
	Organization of cytoplasm	4.77E-04	Increased	2.1	140
	Formation of cellular protrusions	2.71E-03	Increased	2.1	64
	Formation of neurites	8.53E-03	Increased	2.0	12
	Formation of microtubules	9.76E-03	Increased	2.1	11
Cellular development	Immortalization	3.44E-02	Increased	2.2	6
	Differentiation of tumor cell lines	3.73E-02	Increased	2.1	36
Cellular movement	Cell movement of tumor cell lines	2.32E-02	Increased	2.4	62
	Migration of skin cell lines	2.37E-02	Increased	2.2	6
Gene expression	Binding of homeodomain binding site	7.36E-03	Increased	2	4
	Initiation of transcription	2.86E-02	Increased	2.2	12
	Repression of RNA	3.43E-02	Increased	3.2	25
ONS cells					
Cellular movement	Migration of tumor cell lines	1.74E-03	Increased	2.4	58
	Cell movement of brain cancer cell lines	5.32E-03	Increased	2.1	13
	Migration of brain cancer cell lines	1.17E-02	Increased	2.1	11
Cellular growth and proliferation	Proliferation of muscle cells	1.28E-02	Increased	2.4	19
Cell death and survival	Cell death of fibroblast cell lines	1.75E-02	Increased	2.2	65
	Apoptosis of rhabdomyosarcoma cell lines	1.78E-02	Increased	2.2	5
	Cell death of hepatocytes	2.46E-02	Increased	2.2	23
	Cell death of connective tissue cells	2.90E-02	Increased	2.1	66
Organismal Development	Development of blood vessel	4.67E-05	Increased	2.1	93
Cardiovascular System Development & Function	Development of cardiovascular system	6.51E-05	Increased	2.1	126
DNA Replication, Recombination, & Repair	Degradation of DNA	7.59E-03	Decreased	−2.2	27
iPSC-derived neurons^16^					
Cellular Movement	Cell movement of neurons	5.75E-05	Increased	2.7	36
Cell Death	Anoikis of tumor cell lines	9.93E-05	Decreased	−2.6	10

(Top categories ranked according to *p*-value and with a predicted Activation Score, z > 2)

We reanalyzed published transcriptome data from iPS-derived neurons generated from patients and controls.^16^ Raw fluorescence data were obtained from NCBI GEO (Accession # GSE25673). Differentially expressed genes were identified using the same analysis protocols and were then subject to GO analysis (Table 2). The differences between patient-derived and control-derived neurons were in two categories, “cell death” and “cell movement” (Table 2). Pathway analysis of the differentially expressed genes identified one pathway common to the three cell types and the iPS cell-derived neurons^16^ (“axonal guidance”) and several others shared by two of the three cell types and neurons (Supplementary Table 5, showing the pathways passing statistical significance at *P* < 0.05).

### Interactions between DNA methylation and gene expression

**Fig. 3:**
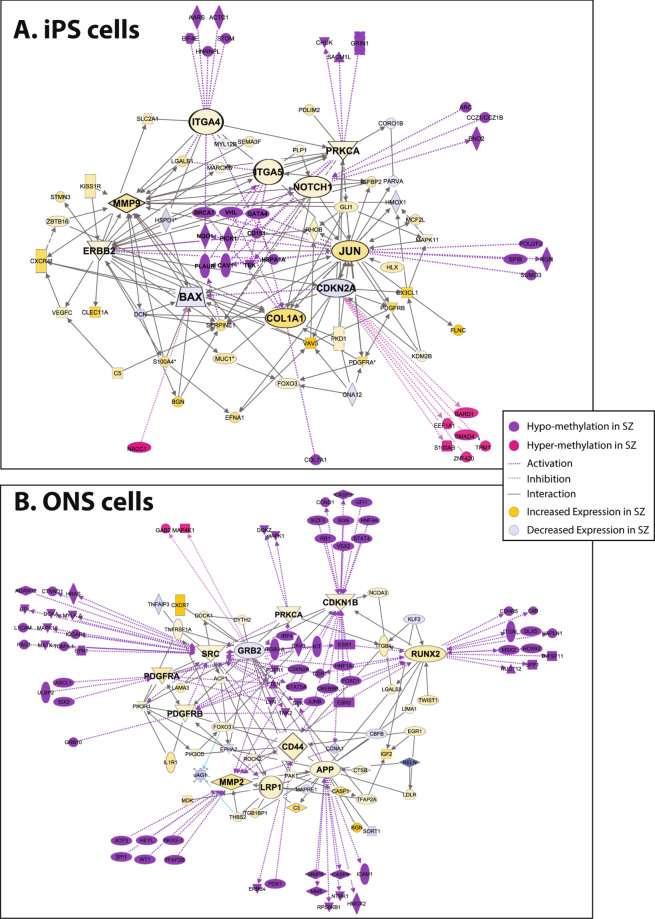
Gene interactions among identified genes in cell movement. Interaction networks constructed from the genes that contributed statistically to the GO category cell movement: **a** iPS cell network; **b** ONS cell network. Differentially expressed genes in patient-derived and control-derived cells and contributing to cell movement (*yellow* and *blue symbols*) were subjected to network analysis based on first-order connections between them (*lines*). *Yellow symbols* are genes with increased expression in patient-derived cells; *blue symbols* are those with decreased expression. The size of the *symbols* represents the magnitude of difference in gene expression between patient-derived and control-derived cells. Differentially methylated gene loci in patient-derived and control-derived cells were then mapped onto the gene expression network (*purple* and *pink symbols*). *Purple symbols* are hypomethylated loci; *pink symbols* are hypermethylated loci. The size of the *symbols* represents the magnitude of difference in DNA methylation between patient-derived and control-derived cells. First-order interactions of identified hypomethylated genes were associated with increased expression of identified genes, whereas hypermethylated genes were associated with decreased expression of identified genes

We looked for a relationship between the differentially DNA methylated loci and the differentially expressed genes in the GO category “cell movement”, which was common to iPS cells and ONS cells for DNA methylation and gene expression. Ingenuity pathway analysis (IPA) was used to construct networks of the differentially methylated and differentially expressed genes in iPS cells and ONS cells. Genes were included if they had direct interactions. Coordination between DNA methylation and gene expression was noted: hypomethylated loci associated with increased gene expression and hypermethylated loci associated with decreased gene expression. In iPS cells, the 49 differentially expressed genes (“nodes” in Fig. 3a) were connected by 248 different interactions (“edges”), with 1–20 edges per node (average 5). In ONS cells, the 59 differentially expressed genes (“nodes” in Fig. 3b) were connected by 374 different interactions (“edges”), with 1–30 edges per node (average 6). In Fig. 3 the size of the gene name is proportional to the number of interactions it has with other genes in the network, demonstrating the relative importance of those genes in the regulatory network. The differentially expressed genes with the greatest number of interactions were also those with the largest numbers of interactions with the differentially methylated genes (*purple* and *pink labels*, Figs. 3a, b). Genes whose expression was increased in patient-derived cells (*yellow labels*, Figs. 3a, b) were interconnected with hypomethylated genes (*purple labels*), whereas genes whose expression was decreased (*blue labels*) were interconnected with hypermethylated genes (*pink labels*).

### Methylated and expressed genes associated with schizophrenia protein-protein interaction (SZ-PPI) network

In order to evaluate either cell-specific DNA methylation or gene expression profiles were associated with schizophrenia risk functional pathways, we used a SZ-PPI network that was built from genes representing confident loci identified by genome-wide association studies,^17^ and their known protein-protein interactions.^18^ There were significant associations with the SZ-PPI network and the differentially methylated genes of ONS cells (*Z*-score = 2.72, *P* = 0.003) and fibroblasts (*Z*-score = 1.94, *P* = 0.03) but not the iPS cells (*Z*-score = 1.06, *p* = 0.1). The differentially expressed genes in iPS cells and ONS cells were significantly associated with the SZ-PPI network (iPS cells: *Z*-score = 12.73, *P* = 2e−37; ONS cells: *Z*-score = 2.77, *P* = 0.003) but not the fibroblasts (*Z*-score = −0.66, *P* = 0.7).

### Expression of the differentially methylated genes is modulated during brain development

**Fig. 4:**
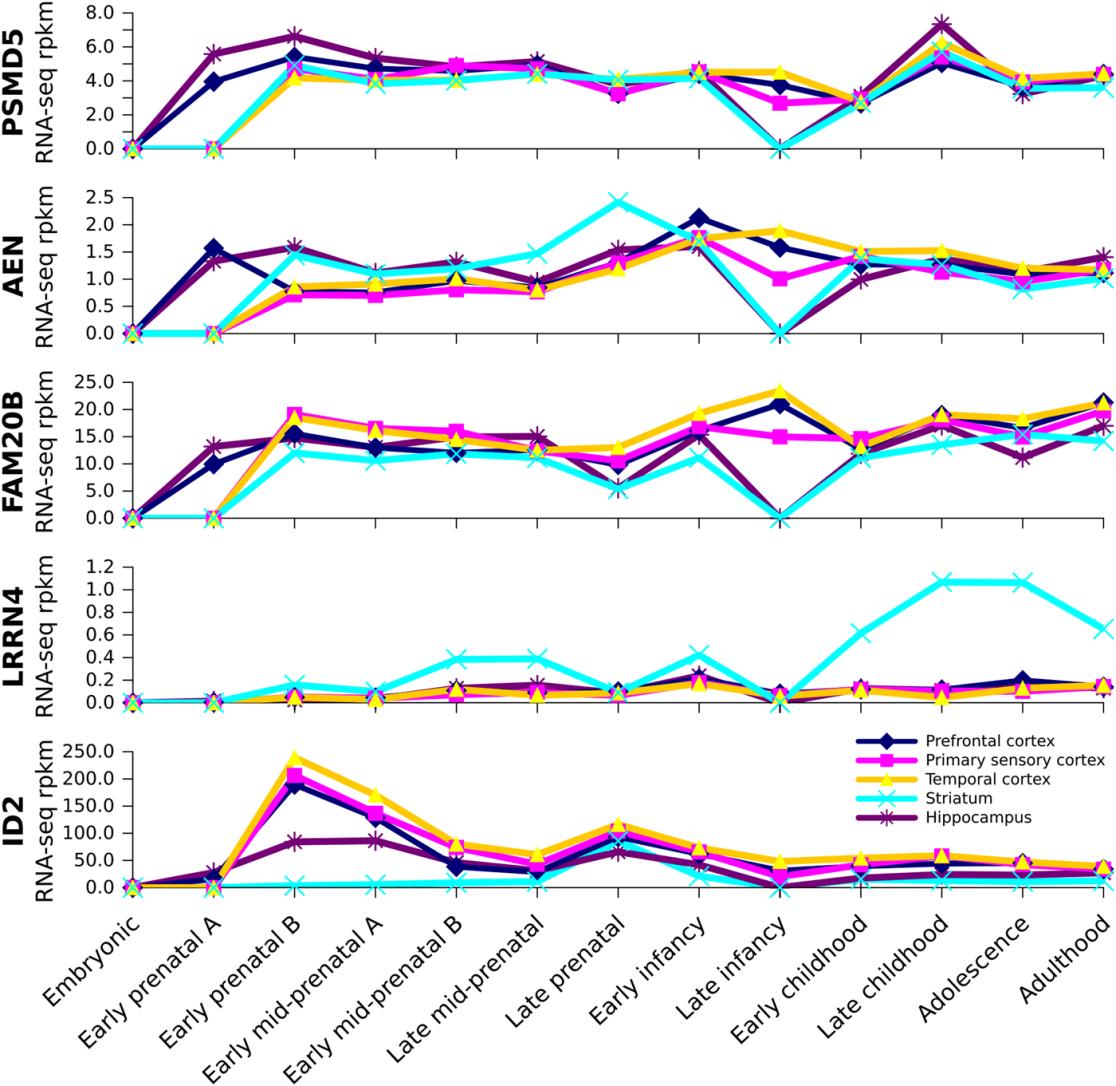
Expression of identified genes in brain at different stages of human development. Brainspan RNA-seq developmental transcriptome data showing developmental profiles of *PSMD5*, *AEN*, *FAM20B*, *LRRN4*, and *ID2*, the genes found to be similarly methylated in patient-derived iPS cells, ONS cells, and fibroblasts compared to control-derived cells. The *x-axis* shows the developmental stages (defined by Brainspan, see Methods). The *y-axis* shows the level of expression in each region at each developmental stage in RPKM (reads per kilobase of exon per million mapped reads)

All five of the differentially methylated genes in the three different schizophrenia-derived cells are expressed in the human brain during development and into adulthood (Fig. 4). Temporal patterns of expression were similar throughout the cortex in contrast to the characteristic expression patterns in hippocampus and striatum. *ID2* is highly expressed in the cortex with peak expression during the early prenatal period (8–9 weeks post conception) decreasing at later periods. *PSMD5*, *AEN*, and *FAM20B* are similar in their developmental patterns of expression from the earliest stage of development (4–7 weeks post conception) after which expression in the cortex does not vary much throughout development. During late infancy there is a transient drop in expression in hippocampus and striatum with cortical expression maintained through to adulthood. *LRRN4* is virtually absent from the cortex and hippocampus; its highest expression is seen in the striatum from late childhood through to adulthood.

## Discussion

Our experiments show that DNA methylation in patient iPS cells is different from control cells. If iPS cells reflect what happens in the embryo, then schizophrenia risk genes may be acting very early in development. The results also show that schizophrenia-associated DNA methylation in iPS cells is not shared by adult cells (ONS cells and fibroblasts) from the same individuals, except for five gene loci that were differentially methylated in all three cell types in patient cells compared to control cells: four hypomethylated genes (*PSMD5*, *AEN*, *FAM20B, LRRN4*) and one hypermethylated gene (*ID2*). *PSMD5*, *AEN* and *FAM20B* shows moderate levels of mRNA expression throughout the cortex at all ages starting very early in development. Hypomethylation would be predicted to enhance these expression levels as observed for *PSMD5*, whose expression is significantly increased in the frontal cortex in postmortem brain in schizophrenia.^19^ *LRRN4* expression is absent from the cortex, but is expressed in the striatum in later stages of development and into adulthood. None of these genes has been previously linked to schizophrenia. *PSMD5* inhibits the proteasome after induction by NFκB;^20^ *LRRN4* is a transmembrane adhesion protein involved in neurite growth;^21^ *FAM20B* is a Golgi-located kinase that regulates the number of glycosaminoglycan chains in proteoglycans;^22^ and *AEN* is an endonuclease controlling autophagy, whose transcription is regulated by the p53 family of genes.^23^ *ID2* mRNA is expressed at very high levels during early prenatal stages after which its expression decreases with age. Hypermethylation of *ID2* would be predicted to blunt this early peak of expression. *ID2* is expressed in neuroblasts and some neurons during embryogenesis^24^ and enhances cell proliferation and regulates axonal growth.^25^ Although these gene loci were methylated in the same way in all three cell types, it is too early to say whether they might be carried as heritable signals. In general though, the lack of overlap among the cell types in schizophrenia-associated DNA methylation suggests that most may be too labile to convey a heritable signal.^5^ These spatial and temporal patterns of gene expression in the developing cortex, combined with schizophrenia-associated DNA methylation, make these genes interesting targets for future research on the neurodevelopmental origins of schizophrenia.

As a way of assessing the link between genetic risk for schizophrenia and DNA methylation we tested the differentially methylated genes for their association with the SZ-PPI network, which is based on schizophrenia risk genes from genome-wide association studies.^18^ The differentially methylated genes were significantly over-represented in the SZ-PPI, indicating that genetic risk and epigenetic modifications converged on the same regulatory network, possibly driving the association of the network with the differentially expressed genes. These associations were most consistent for ONS cells. In all three cell types there was convergence of schizophrenia-associated DNA methylation and gene expression onto cell functions important for brain development (GO categories “Cellular Movement”, “Cellular Growth and Differentiation” and “Cell Death and Survival”) even though the genes contributing to these functions were different and largely unique for each cell type. “Cellular Movement” is particularly interesting because cell migration is dysregulated in patient-derived ONS cells compared to control-derived cells.^26, 27^ Three of the genes with shared DNA methylation status could affect adhesion and motility: *LRRN4* and *ID2* are involved with NOGO cell adhesion receptor signaling^21, 25^ and *FAM20B* is involved in cell adhesion by regulating proteoglycan composition.^22^ Cell movement was also impaired in patient-derived B lymphoblasts compared to controls.^28^ Dysregulated cell motility and migration may be a general property of cells derived from schizophrenia patients as many schizophrenia risk genes are over-represented in pathways controlling neuronal migration and cell adhesion.^29–31^ Altered cell migration is a plausible risk for altering neurodevelopmental trajectories in schizophrenia.^32, 33^

DNA methylation analysis of blood DNA from 98 patients with schizophrenia and 102 controls revealed 16 CpG loci that were significantly associated with schizophrenia with the inflammatory response as the most significant biological function affected in the patient-derived DNA.^34^ Similarly, pathway analysis of DNA methylation in blood from twins discordant for schizophrenia identified differentially methylated genes associated with “hematological system development and function”.^35^ Taken together with our observations, it seems reasonable to conclude that schizophrenia-associated DNA methylation and downstream gene expression are dominated by the cell type and its functional demands.

This study provides a genome-wide view of schizophrenia-associated DNA methylation (and gene expression) of three cell types: iPS cells, in which the reprogramming process erases and rewrites the epigenome, and ONS cells and fibroblasts, which may carry epigenetic marks from the adults from which they derive. The epigenetic marks on different cell types from the same individuals show that schizophrenia status affects each cell type differently. How this would be reflected in the developing brain remains to be explored and the effect of schizophrenia-associated DNA methylation on neurons and glia is unknown. Additionally, there are other epigenetic mechanisms that may contribute. For example, histone methylation in patient-derived primary olfactory cells identified schizophrenia-associated histone methylation affecting 22 genes that contribute to cell functions different from those identified here.^36^

### Technical issues

All patients were medicated with antipsychotic drugs and all smoked cigarettes at the time of biopsy, whereas none of the controls did. The medications and doses differed among the patients. All cells were cultured for more than 4 weeks, so any direct drug effects would be eliminated, but it is possible that medication and smoking prior to tissue biopsy of nose and skin could lead to schizophrenia-associated DNA methylation. This seems unlikely given the small overlap among the cell types, assuming that medication-induced or smoking-induced DNA methylation would likely be targeted to specific genes. In previous analyses of the same ONS cells and fibroblasts we found no patient-control differences in gene expression or cell functions that can be ascribed to medication or smoking.^15^

Our findings should be considered preliminary, being based on only a small number of individuals. While each is represented by three cell types, these relatively small numbers make the results open to selection bias and individuals were not represented equally in the numbers of cell lines because of technical difficulties. Small sample sizes obscure group differences because of individual variability among the patients and controls. Variability was minimized where possible by using standardized culture protocols, and the iPS cells were selected for homogeneity using the pluripotency marker, SSEA4.^37^ The small number of individuals limits the statistical power of the study, leading to underestimation of the number of schizophrenia-associated DNA methylated loci at the expense of the reduction in false positives. It can be expected that more loci will emerge as larger samples are examined. These statistical limitations are in common with other patient-derived iPS cell studies because the difficulties of iPS cell generation and maintenance impart restraints on the number of cell lines that are feasible to generate and experiment upon.^37^

## Methods

### Patient-derived and control-derived cell lines

The patients and controls from whom the cells were derived are a subset of a cohort of 18 donors from whom ONS cell gene expression and cell functions are previously described.^15, 26, 38, 39^ Demographic details of the individuals and details of the cell lines derived from them are in Table 3. Nasal and skin biopsy procedures were approved by the Ethics Committee for West Moreton Region, Queensland Health, and the Griffith University Human Ethics Committee. As part of the approval process, all participants gave written, informed consent for their cells to be grown in vitro, banked, and used for experiments to understand the biological bases of schizophrenia. The approved biopsy procedure and subsequent experiments were conducted according to the guidelines of the National Health and Medical Research Council of Australia. The iPS cell lines were a subset selected from our collection characterized previously and considered to be fully reprogrammed by multiple criteria.^37^

**Table 3:** Participants and cell lines

Patient ID	Status	Sex	Age	Smoker	Medication	iPS Cell ID	Passage	ONS Cell ID	Fibroblast ID
100020002	Control	M	47	N	Nil	GU9563i-1	n.a.	GU9563-ONS	GU9563-Fb
100020003	Control	M	28	N	Nil	GU9565i-1	16	GU9565-ONS	GU9565-Fb
						GU9565i-2	18		
						GU9565i-2	22		
100030004	Control	M	46	N	Nil	GU9569-L2	19	GU9569-ONS	GU9569-Fb
100030007	Control	M	51	N	Nil^a^	GU9575-ASC2	19		GU9572-Fb
300020003	Patient	M	21	Y	Quetiapine^b^	GU8063i-sz1	23	GU8063-ONS	GU8063-Fb
300020005	Patient	M	49	Y	Clozapine	GU8067i-4	38	GU8067-ONS	GU8067-Fb
						GU8067i-B1	20		
300020007	Patient	M	44	Y	Clozapine^c^	GU8069-3	29	GU8069-ONS	GU8069-Fb
						GU8069-B1	19		
300020008	Patient	M	28	Y	Flupenthixol decanoate	GU8070-8	34	GU8070-ONS	GU8070-Fb
						GU8070-8	40		

n.a. not available

^a^Other medications: metformin hydrochloride, sotalol hydrochloride, hydrochlorothiazide; irbesartan, digoxin

^b^Other medications: paroxetine

^c^Other medications: lithium carbonate, atenolol

### Cell culture

Aliquots of established ONS cells and fibroblasts frozen in liquid nitrogen were thawed and grown under standard conditions on tissue culture plastic in Dulbecco’s Minimum Essential Medium with F12 (DMEM-F12; JRH Biosciences) supplemented with 10% fetal bovine serum (Gibco BRL) at 37 °C and 5% CO_2_.^15^ At each passage, and for DNA and mRNA extraction, the cells were dissociated with trypsin. Established iPSC lines^37^ were grown on a monolayer of mouse embryonic fibroblasts (12,000 cells/cm^2^). The culture medium consisted of DMEM-F12 with: 20% Knockout Serum Replacement, 2 mM GlutaMAX-1, 1× Non-Essential Amino Acids, 0.1 mM β-mercaptoethanol, 1× penicillin/streptomycin (all from Invitrogen, Carlsbad, CA), and 50 ng/ml basic fibroblast growth factor (FGF2; Millipore). Cell lines were passaged mechanically. The cell lines were characterized previously and shown to be pluripotent.^37^ A human embryonic stem cell line (HES3) was used as a comparator control. For the DNA methylation and gene expression analyses, the pluripotent fractions of the iPS cells and HES3 cell cultures were selected by flow cytometry using the cell surface marker, SSEA4. The cultures were harvested using collagenase IV, washed in DMEM-F12 and immunostained with SSEA4 (MAB4304; Millipore), and then sorted using flow cytometry (FACSAria flow cytometer; BD Biosciences, San Diego, CA, http://www.bdbiosciences.com). This sorted SSEA4 fraction was used for subsequent DNA and RNA isolation and analysis. The cells used here are available from the Griffith Institute for Drug Discovery, Griffith University, Australia (http://www.griffith.edu.au/), subject to patient consent, an appropriate material transfer agreement, and payment of shipping and handling fees.

### Bisulfite conversion

Genomic DNA was extracted from several passages from each cell line with a Wizard^®^ Genomic DNA Purification Kit (Promega) according to the manufacturer’s protocol. DNA quantity and quality were determined using Quant-iT^™^ PicoGreen^®^ dsDNA Kits (Invitrogen). Sodium bisulfite conversion of 500 ng genomic DNA was performed with an EZ DNA Methylation Kit (Zymo Research), with the alternative incubation conditions recommended for Illumina Infinium^®^ Methylation Assay. After conversion, DNA quantity was measured using a NanoDrop spectrophotometer set at ssDNA and percentage recovery calculated.

### DNA methylation profiling

Methylation profiles were generated with the Infinium HumanMethylation27 BeadChip Kit (Illumina, San Diego, CA, USA), according to the manufacturer’s protocol. Raw data were imported in GenomeStudio, with which the fluorescence intensity of each probe was extracted and used to generate *β*-values: methylated intensity divided by the sum of methylated and unmethylated intensity. For general assessment of the methylation state of each cell type, probes were binned into three categories: hypermethylated ≥70%, hypomethylated ≤30%, mid-methylated 30–70%. The *β*-value was used for initial clustering because it is biologically more intuitive; however, it is severely limited for statistical analysis because of heteroscedasticity outside the middle range.^40^ For all statistical comparisons the fluorescence intensities were converted to *M*-value using the lumi package in R. Methylation data were deposited into ArrayExpress (Accession Number E-MTAB-5016).

### Gene expression profiling

RNA was extracted from cell lysates using the RNeasy Micro Kit (Qiagen) according to the manufacturer’s protocol. For comparison of gene expression and DNA methylation in the cells from the same individuals the gene expression profiles of the selected iPS cells, ONS cells, and fibroblasts were analyzed using lumi and limma packages in R/BioConductor.^37^ The gene expression data comparing the three cell types are deposited into ArrayExpress (Accession Number E-TABM-5016). For comparison of gene expression in the three cell types the same iPS cell gene expression data were used, while the ONS cell and fibroblast gene expression was from the larger data set that included the same individuals,^15^ available at ArrayExpress accession number E-TABM-724.

### Expression of selected genes in human brain during development

We identified five genes whose DNA methylation in all three patient cell types was different from controls. The expression of these genes was investigated in the developing human brain using available RNA sequencing data from BrainSpan database (http://www.brainspan.org).^41^ This is a database of RNA expression (RNA-seq) of about 52,400 genes in multiple brain regions generated from 579 tissue samples from 41 developing and adult postmortem brains’ early prenatal development to adulthood, divided into 13 developmental stages: embryonic [4–7 post-conceptional weeks (pcw)]; early prenatal A (8–9 pcw); early prenatal B (10–12 pcw); early mid-prenatal A (13–15 pcw); early mid-prenatal B (16–18 pcw); late mid-prenatal (19–24 pcw); late prenatal (25–38 pcw); early infancy (birth to 5 months); late infancy (6–18 months); early childhood (19 months to 5 years); late childhood (6–11 years); adolescence (12–19 years); adulthood (20–60+years). In the present study, the cortical region data were grouped into Prefrontal cortex, including ventrolateral prefrontal cortex, orbitofrontal cortex, medial prefrontal cortex (anterior cingulate), and dorsolateral prefrontal cortex; primary sensory cortex, including primary motor cortex (area M1, area 4), primary auditory cortex, primary visual cortex (striate cortex, area V1/17), and primary somatosensory cortex (area S1, areas 3, 1, 2); temporal cortex, including posterior superior temporal cortex (area 22c) and inferolateral temporal cortex (area 20). Subcortical regions chosen were the striatum and hippocampus. Brainspan provides quantitative data based on RNA-seq expression in the commonly used units of RPKM (reads per kilobase of exon model per million mapped reads). For details see the “Technical White Paper: Transcriptome profiling by RNA sequencing and Exon Microarray” (available at http://www.brainspan.org).

### Statistical analysis

DNA methylation and gene expression profiles for each control-derived cell type (iPS cells, ONS cells, and fibroblasts) and the ES cells were subjected to principal components analysis (PCA) (princomp package in R) using all the probes detected on each array. To eliminate cell culture artefacts and technical errors affecting methylation status, all samples were measured in triplicate. PCA identified one outlier clustering separately: GU9569fb-cont P6 (Supplementary Figure 1) that was removed from the analysis. All correlation coefficients (*r* ^2^) between replicates were very high (average 0.99, range 0.97–0.99). PCA provided an unbiased method to determine whether the cell types could be distinguished from each other. Cell-type differences in gene expression and methylation were determined with limma package in R. For the analysis, the consecutive passages were treated as technical replicates and accounted for in the linear model. The iPS cell line clones of each patient were treated as biological replicates. All *p*-values were adjusted for multiple testing using the Benjamini and Hochberg procedure for FDR correction. The differentially methylated and differentially expressed genes were then subjected to cluster analysis using Cluster 3.0^42^ and visualized in Java Treeview.^43^

Using IPA 8.5 (Ingenuity Systems, Redwood, CA), the differentially methylated and expressed genes were subjected to GO over-representation analysis,^44^ using right-tailed Fisher’s exact test with Benjamini and Hochberg multiple testing correction. For the differentially methylated genes, GO categories for each cell type were ranked according to probability. For the differentially expressed genes, GO categories were further filtered according to predicted activation state, which is calculated using the IPA *Z*-score algorithm that predicts the direction of change for the function. An absolute *z*-score of ≥2 was considered significant. IPA was used to build networks between differentially expressed genes (shown as nodes) and interactions between them (shown as connecting lines, known as edges). Edges between differentially methylated genes and differentially expressed genes were only considered if there was evidence of activation (hypomethylation of CpG loci leading to up-regulated gene expression) or inhibition (hyper-methylation of CpG loci leading to down-regulated gene expression).

The methylome and transcriptome data from iPS cells, ONS cells, and fibroblasts were compared to a SZ-PPI network^18^ built from ‘seed’ genes and their first-degree interacting neighbors based on high confidence loci identified in previous genome-wide association studies obtained from the schizophrenia gene resource.^17^ The standardized *Z*-score was estimated using a binomial distribution with a one-tailed *Z*-test (Eq. 1) at *P* < 0.05 with critical *Z*-score of 1.65.

1 $$Z=\frac{O-E}{\sqrt{(N-1)pq}}$$

where: *O* = observed number of gene variants in the SZ-PPI network, *E* = expected number of gene variants in the SZ-PPI network, *N* = total number of gene variants, *p* = expected frequency of gene variants, *q* = 1−p

## Electronic supplementary material

Supplementary Table 1

Supplementary Figure 1

Supplementary Table 2

Supplementary Table 3

Supplementary Table 4

Supplementary Table 5
