# Supplementary material for: DNA methylation in schizophrenia in different patient-derived cell types
Source: NPJ Schizophr. 2017 Jan 23;3:6. doi: 10.1038/s41537-016-0006-0 (PMC5441549; doi:10.1038/s41537-016-0006-0)
Supplement: Supplementary file 5 — Supplementary Table 4 [file 41537_2016_6_MOESM5_ESM.docx]

**Vitale et al, Supplementary Table 4**

| This table lists the results of the statistical analysis showing the five gene loci whose relative methylation status is shared by all three cell types | | | | | | | | |
| --- | --- | --- | --- | --- | --- | --- | --- | --- |
| (iPS cells, ONS cells, fibroblasts). | | |  |  |  |  |  |  |
|  |  |  |  |  |  |  |  |  |
| Columns show p-value adjusted for multiple testing (Benjamini and Hochberg False Discovery Rate Correction) and Fold Change | | | | | | | |  |
| Positive means patient cells are hypermethylated compared to control cells; negative means patient cells are hypomethylated compared to control cells. | | | | | | | | |
| **SYMBOL** | **iPS Sz Vs Control B & H Adj. P-Value** | **iPS Sz Vs Control log Fold Change** | **ONS Sz Vs Control B & H Adj. P-Value** | **ONS Sz Vs Control log Fold Change** | **Fibro Sz Vs Control B & H Adj. P-Value** | **Fibro Sz Vs Control log Fold Change** | **PRODUCT** |  |
| *PSMD5* | 2.21E-13 | -3.1473 | 3.16E-07 | -2.33589 | 2.6E-07 | -2.13554 | proteasome 26S non-ATPase subunit 5 |  |
| *PSMD5* | 1.26E-08 | -2.85594 | 0.002765 | -1.68128 | 0.001128 | -1.71368 | proteasome 26S non-ATPase subunit 5 |  |
| *ID2* | 0.000624 | 1.463368 | 0.000102 | 1.879459 | 0.001221 | 1.469497 | inhibitor of DNA binding 2 |  |
| *LRRN4/C20orf75* | 0.000913 | -2.03543 | 0.037081 | -1.46865 | 0.002853 | -1.85435 | hypothetical protein LOC164312 |  |
| *FAM20B* | 0.004043 | -0.84524 | 8.76E-05 | -1.3588 | 0.018614 | -0.80651 | family with sequence similarity 20; member B |  |
| *AEN/ISG20L1* | 0.012523 | -0.6455 | 0.000144 | -1.1606 | 0.011697 | -0.75272 | interferon stimulated exonuclease gene 20kDa-like 1 |  |
