# Supplementary material for: DNA methylation in schizophrenia in different patient-derived cell types
Source: NPJ Schizophr. 2017 Jan 23;3:6. doi: 10.1038/s41537-016-0006-0 (PMC5441549; doi:10.1038/s41537-016-0006-0)
Supplement: Supplementary file 6 — Supplementary Table 5 [file 41537_2016_6_MOESM6_ESM.docx]

| **Vitale et al, Supplementary Table 5.** Ingenuity Pathway Analysis of DNA methylation and gene expression | | | | | |  |  |  |  |  |  |  |
| --- | --- | --- | --- | --- | --- | --- | --- | --- | --- | --- | --- | --- |
|  |  |  |  |  |  |  |  |  |  |  |  |  |
|  | **Key** |  |  |  |  |  |  |  |  |  |  |  |
|  |  | All cell types | |  |  |  |  |  |  |  |  |  |
|  |  | ONS cells and fibroblasts | |  |  |  |  |  |  |  |  |  |
|  |  | ONS cells and neurons | |  |  |  |  |  |  |  |  |  |
|  |  | iPS cells and neurons | |  |  |  |  |  |  |  |  |  |
|  |  | iPS cells and fibroblasts | |  |  |  |  |  |  |  |  |  |
|  |  | iPS cells, fibroblasts and neurons | |  |  |  |  |  |  |  |  |  |
|  |  |  |  |  |  |  |  |  |  |  |  |  |
| **DNA Methylation pathways** |  |  |  |  |  |  |  |  |  |  |  |  |
| **ONS cells** | **p-value** | **No of genes** |  |  |  |  |  |  |  |  |  |  |
| 3-phosphoinositide Degradation | 0.035481 | 6 |  |  |  |  |  |  |  |  |  |  |
| Actin Cytoskeleton Signaling | 0.040738 | 19 |  |  |  |  |  |  |  |  |  |  |
| Acute Phase Response Signaling | 0.012589 | 18 |  |  |  |  |  |  |  |  |  |  |
| AMPK Signaling | 0.01122 | 15 |  |  |  |  |  |  |  |  |  |  |
| Androgen Biosynthesis | 0.032359 | 3 |  |  |  |  |  |  |  |  |  |  |
| Aryl Hydrocarbon Receptor Signaling | 0.042658 | 14 |  |  |  |  |  |  |  |  |  |  |
| Bladder Cancer Signaling | 0.00195 | 13 |  |  |  |  |  |  |  |  |  |  |
| CDK5 Signaling | 0.037154 | 10 |  |  |  |  |  |  |  |  |  |  |
| Colanic Acid Building Blocks Biosynthesis | 0.039811 | 3 |  |  |  |  |  |  |  |  |  |  |
| Dopamine Degradation | 0.046774 | 4 |  |  |  |  |  |  |  |  |  |  |
| Dopamine Receptor Signaling | 0.014791 | 10 |  |  |  |  |  |  |  |  |  |  |
| eNOS Signaling | 0.024547 | 14 |  |  |  |  |  |  |  |  |  |  |
| ERK5 Signaling | 0.035481 | 8 |  |  |  |  |  |  |  |  |  |  |
| Estrogen-Dependent Breast Cancer Signaling | 0.0302 | 9 |  |  |  |  |  |  |  |  |  |  |
| Estrogen-mediated S-phase Entry | 0.00257 | 6 |  |  |  |  |  |  |  |  |  |  |
| Fc Epsilon RI Signaling | 0.026303 | 12 |  |  |  |  |  |  |  |  |  |  |
| Fcγ Receptor-mediated Phagocytosis in Macrophages and Monocytes | 0.042658 | 10 |  |  |  |  |  |  |  |  |  |  |
| FXR/RXR Activation | 0.014454 | 11 |  |  |  |  |  |  |  |  |  |  |
| Glioma Signaling | 0.012023 | 12 |  |  |  |  |  |  |  |  |  |  |
| Gluconeogenesis I | 0.013183 | 5 |  |  |  |  |  |  |  |  |  |  |
| G-Protein Coupled Receptor Signaling | 0.000209 | 50 |  |  |  |  |  |  |  |  |  |  |
| Hepatic Cholestasis | 0.001698 | 18 |  |  |  |  |  |  |  |  |  |  |
| Hepatic Fibrosis / Hepatic Stellate Cell Activation | 0.00182 | 18 |  |  |  |  |  |  |  |  |  |  |
| Human Embryonic Stem Cell Pluripotency | 0.020417 | 14 |  |  |  |  |  |  |  |  |  |  |
| IL-8 Signaling | 0.027542 | 18 |  |  |  |  |  |  |  |  |  |  |
| Leukocyte Extravasation Signaling | 0.004677 | 21 |  |  |  |  |  |  |  |  |  |  |
| Leukotriene Biosynthesis | 0.006166 | 4 |  |  |  |  |  |  |  |  |  |  |
| LPS/IL-1 Mediated Inhibition of RXR Function | 0.041687 | 20 |  |  |  |  |  |  |  |  |  |  |
| LXR/RXR Activation | 0.000355 | 18 |  |  |  |  |  |  |  |  |  |  |
| Maturity Onset Diabetes of Young (MODY) Signaling | 0.040738 | 4 |  |  |  |  |  |  |  |  |  |  |
| Neuroprotective Role of THOP1 in Alzheimer's Disease | 0.009333 | 7 |  |  |  |  |  |  |  |  |  |  |
| NF-κB Signaling | 0.046774 | 16 |  |  |  |  |  |  |  |  |  |  |
| p53 Signaling | 0.00138 | 14 |  |  |  |  |  |  |  |  |  |  |
| p70S6K Signaling | 0.012882 | 14 |  |  |  |  |  |  |  |  |  |  |
| PI3K/AKT Signaling | 0.031623 | 13 |  |  |  |  |  |  |  |  |  |  |
| Prostate Cancer Signaling | 0.026303 | 10 |  |  |  |  |  |  |  |  |  |  |
| PTEN Signaling | 0.001514 | 16 |  |  |  |  |  |  |  |  |  |  |
| Regulation of Actin-based Motility by Rho | 0.039811 | 9 |  |  |  |  |  |  |  |  |  |  |
| Role of Pattern Recognition Receptors in Recognition of Bacteria and Viruses | 0.026303 | 11 |  |  |  |  |  |  |  |  |  |  |
| Serine Biosynthesis | 0.020417 | 2 |  |  |  |  |  |  |  |  |  |  |
| Serotonin Receptor Signaling | 0.037154 | 5 |  |  |  |  |  |  |  |  |  |  |
| Thyroid Cancer Signaling | 0.009333 | 7 |  |  |  |  |  |  |  |  |  |  |
| VDR/RXR Activation | 0.019055 | 10 |  |  |  |  |  |  |  |  |  |  |
| Virus Entry via Endocytic Pathways | 0.018197 | 11 |  |  |  |  |  |  |  |  |  |  |
| γ-glutamyl Cycle | 0.025119 | 3 |  |  |  |  |  |  |  |  |  |  |
|  |  |  |  |  |  |  |  |  |  |  |  |  |
| **iPS cells** | **p-value** | **No of genes** | |  |  |  |  |  |  |  |  |  |
| Embryonic Stem Cell Differentiation into Cardiac Lineages | 0.008128 | 2 |  |  |  |  |  |  |  |  |  |  |
| Fatty Acid Biosynthesis Initiation II | 0.027542 | 1 |  |  |  |  |  |  |  |  |  |  |
| IL-1 Signaling | 0.037154 | 4 |  |  |  |  |  |  |  |  |  |  |
| LPS/IL-1 Mediated Inhibition of RXR Function | 0.033884 | 7 |  |  |  |  |  |  |  |  |  |  |
| Palmitate Biosynthesis I (Animals) | 0.041687 | 1 |  |  |  |  |  |  |  |  |  |  |
| Pyruvate Fermentation to Lactate | 0.041687 | 1 |  |  |  |  |  |  |  |  |  |  |
| RhoGDI Signaling | 0.00182 | 8 |  |  |  |  |  |  |  |  |  |  |
| Serotonin Degradation | 0.041687 | 3 |  |  |  |  |  |  |  |  |  |  |
| Signaling by Rho Family GTPases | 0.032359 | 7 |  |  |  |  |  |  |  |  |  |  |
| Sulfate Activation for Sulfonation | 0.027542 | 1 |  |  |  |  |  |  |  |  |  |  |
| Wnt/β-catenin Signaling | 0.02884 | 6 |  |  |  |  |  |  |  |  |  |  |
|  |  |  |  |  |  |  |  |  |  |  |  |  |
| **Fibroblasts** | **p-value** | **No of genes** | |  |  |  |  |  |  |  |  |  |
| 14-3-3-mediated Signaling | 0.020417 | 10 |  |  |  |  |  |  |  |  |  |  |
| 2-amino-3-carboxymuconate Semialdehyde Degradation to Glutaryl-CoA | 0.042658 | 1 |  |  |  |  |  |  |  |  |  |  |
| Acute Phase Response Signaling | 0.005248 | 15 |  |  |  |  |  |  |  |  |  |  |
| AMPK Signaling | 0.000398 | 15 |  |  |  |  |  |  |  |  |  |  |
| Amyotrophic Lateral Sclerosis Signaling | 0.022909 | 9 |  |  |  |  |  |  |  |  |  |  |
| Aryl Hydrocarbon Receptor Signaling | 0.006607 | 13 |  |  |  |  |  |  |  |  |  |  |
| Calcium Signaling | 0.035481 | 13 |  |  |  |  |  |  |  |  |  |  |
| Cardiac Hypertrophy Signaling | 0.025704 | 16 |  |  |  |  |  |  |  |  |  |  |
| Cardiomyocyte Differentiation via BMP Receptors | 0.00631 | 4 |  |  |  |  |  |  |  |  |  |  |
| Coenzyme A Biosynthesis | 0.042658 | 1 |  |  |  |  |  |  |  |  |  |  |
| Death Receptor Signaling | 0.011482 | 7 |  |  |  |  |  |  |  |  |  |  |
| Dendritic Cell Maturation | 0.000151 | 19 |  |  |  |  |  |  |  |  |  |  |
| eNOS Signaling | 0.00912 | 12 |  |  |  |  |  |  |  |  |  |  |
| Gap Junction Signaling | 0.002455 | 15 |  |  |  |  |  |  |  |  |  |  |
| Glutathione-mediated Detoxification | 0.0302 | 4 |  |  |  |  |  |  |  |  |  |  |
| G-Protein Coupled Receptor Signaling | 0.002399 | 37 |  |  |  |  |  |  |  |  |  |  |
| Hepatic Cholestasis | 0.002344 | 14 |  |  |  |  |  |  |  |  |  |  |
| Hepatic Fibrosis / Hepatic Stellate Cell Activation | 0.006607 | 13 |  |  |  |  |  |  |  |  |  |  |
| IL-10 Signaling | 0.008128 | 8 |  |  |  |  |  |  |  |  |  |  |
| IL-12 Signaling and Production in Macrophages | 0.004266 | 13 |  |  |  |  |  |  |  |  |  |  |
| IL-6 Signaling | 0.026915 | 10 |  |  |  |  |  |  |  |  |  |  |
| Leptin Signaling in Obesity | 0.01349 | 8 |  |  |  |  |  |  |  |  |  |  |
| LPS/IL-1 Mediated Inhibition of RXR Function | 0.000437 | 21 |  |  |  |  |  |  |  |  |  |  |
| LPS-stimulated MAPK Signaling | 0.035481 | 7 |  |  |  |  |  |  |  |  |  |  |
| LXR/RXR Activation | 1.48E-05 | 17 |  |  |  |  |  |  |  |  |  |  |
| MSP-RON Signaling Pathway | 0.041687 | 5 |  |  |  |  |  |  |  |  |  |  |
| Myc Mediated Apoptosis Signaling | 0.036308 | 6 |  |  |  |  |  |  |  |  |  |  |
| Neuropathic Pain Signaling In Dorsal Horn Neurons | 0.026915 | 9 |  |  |  |  |  |  |  |  |  |  |
| NF-κB Signaling | 0.005248 | 15 |  |  |  |  |  |  |  |  |  |  |
| p53 Signaling | 0.017783 | 9 |  |  |  |  |  |  |  |  |  |  |
| p70S6K Signaling | 0.012882 | 11 |  |  |  |  |  |  |  |  |  |  |
| Phototransduction Pathway | 0.019055 | 6 |  |  |  |  |  |  |  |  |  |  |
| PPAR Signaling | 0.019055 | 9 |  |  |  |  |  |  |  |  |  |  |
| PXR/RXR Activation | 0.002089 | 9 |  |  |  |  |  |  |  |  |  |  |
| RAR Activation | 0.034674 | 13 |  |  |  |  |  |  |  |  |  |  |
| Retinoic acid Mediated Apoptosis Signaling | 0.036308 | 6 |  |  |  |  |  |  |  |  |  |  |
| Serotonin Receptor Signaling | 0.00955 | 5 |  |  |  |  |  |  |  |  |  |  |
| Small Cell Lung Cancer Signaling | 0.031623 | 7 |  |  |  |  |  |  |  |  |  |  |
| The Visual Cycle | 0.023988 | 3 |  |  |  |  |  |  |  |  |  |  |
| Thrombin Signaling | 0.027542 | 14 |  |  |  |  |  |  |  |  |  |  |
| TR/RXR Activation | 0.028184 | 8 |  |  |  |  |  |  |  |  |  |  |
| Type II Diabetes Mellitus Signaling | 0.01 | 11 |  |  |  |  |  |  |  |  |  |  |
| VDR/RXR Activation | 0.048978 | 7 |  |  |  |  |  |  |  |  |  |  |
| VEGF Family Ligand-Receptor Interactions | 0.035481 | 7 |  |  |  |  |  |  |  |  |  |  |
| Xenobiotic Metabolism Signaling | 0.019055 | 20 |  |  |  |  |  |  |  |  |  |  |
|  |  |  |  |  |  |  |  |  |  |  |  |  |
|  |  |  |  |  |  |  |  |  |  |  |  |  |
| **Gene expression pathways** |  |  |  |  |  |  |  |  |  |  |  |  |
| **ONS cells** | **p-value** | **No of genes** | |  |  |  |  |  |  |  |  |  |
| Acute Myeloid Leukemia Signaling | 0.04 | 11 |  |  |  |  |  |  |  |  |  |  |
| Acute Phase Response Signaling | 0.04 | 20 |  |  |  |  |  |  |  |  |  |  |
| Airway Pathology in Chronic Obstructive Pulmonary Disease | 0.02 | 3 |  |  |  |  |  |  |  |  |  |  |
| Axonal Guidance Signaling | 0.04 | 40 |  |  |  |  |  |  |  |  |  |  |
| Butanoate Metabolism | 0.004 | 11 |  |  |  |  |  |  |  |  |  |  |
| GABA Receptor Signaling | 0.03 | 8 |  |  |  |  |  |  |  |  |  |  |
| Hepatic Fibrosis / Hepatic Stellate Cell Activation | 0.0004 | 23 |  |  |  |  |  |  |  |  |  |  |
| Human Embryonic Stem Cell Pluripotency | 0.04 | 16 |  |  |  |  |  |  |  |  |  |  |
| Lysine Degradation | 0.01 | 10 |  |  |  |  |  |  |  |  |  |  |
| Mouse Embryonic Stem Cell Pluripotency | 0.0030 | 16 |  |  |  |  |  |  |  |  |  |  |
| Neuroprotective Role of THOP1 in Alzheimer's Disease | 0.04 | 7 |  |  |  |  |  |  |  |  |  |  |
| Propanoate Metabolism | 0.02 | 9 |  |  |  |  |  |  |  |  |  |  |
| Pyruvate Metabolism | 0.03 | 10 |  |  |  |  |  |  |  |  |  |  |
| Role of IL-17A in Arthritis | 0.02 | 9 |  |  |  |  |  |  |  |  |  |  |
| Role of Macrophages, Fibroblasts and Endothelial Cells in Rheumatoid Arthritis | 0.04 | 31 |  |  |  |  |  |  |  |  |  |  |
| Role of Tissue Factor in Cancer | 0.04 | 14 |  |  |  |  |  |  |  |  |  |  |
| Toll-like Receptor Signaling | 0.04 | 8 |  |  |  |  |  |  |  |  |  |  |
| Valine, Leucine and Isoleucine Degradation | 0.006 | 11 |  |  |  |  |  |  |  |  |  |  |
| VDR/RXR Activation | 0.04 | 11 |  |  |  |  |  |  |  |  |  |  |
| Wnt/β-catenin Signaling | 0.005 | 23 |  |  |  |  |  |  |  |  |  |  |
| β-alanine Metabolism | 0.04 | 7 |  |  |  |  |  |  |  |  |  |  |
|  |  |  |  |  |  |  |  |  |  |  |  |  |
| **iPS cells** | **p-value** | **No of genes** | |  |  |  |  |  |  |  |  |  |
| Actin Nucleation by ARP-WASP Complex | 0.04 | 10 |  |  |  |  |  |  |  |  |  |  |
| Aminosugars Metabolism | 0.0003 | 17 |  |  |  |  |  |  |  |  |  |  |
| Androgen Signaling | 0.004 | 20 |  |  |  |  |  |  |  |  |  |  |
| April Mediated Signaling | 0.03 | 8 |  |  |  |  |  |  |  |  |  |  |
| Axonal Guidance Signaling | 0.008 | 52 |  |  |  |  |  |  |  |  |  |  |
| B Cell Activating Factor Signaling | 0.04 | 8 |  |  |  |  |  |  |  |  |  |  |
| Cardiac β-adrenergic Signaling | 0.02 | 21 |  |  |  |  |  |  |  |  |  |  |
| Caveolar-mediated Endocytosis Signaling | 0.04 | 12 |  |  |  |  |  |  |  |  |  |  |
| CCR5 Signaling in Macrophages | 0.03 | 11 |  |  |  |  |  |  |  |  |  |  |
| CXCR4 Signaling | 0.03 | 22 |  |  |  |  |  |  |  |  |  |  |
| Dopamine Receptor Signaling | 0.01 | 14 |  |  |  |  |  |  |  |  |  |  |
| G Beta Gamma Signaling | 0.04 | 14 |  |  |  |  |  |  |  |  |  |  |
| Gα12/13 Signaling | 0.03 | 18 |  |  |  |  |  |  |  |  |  |  |
| Hepatic Cholestasis | 0.04 | 20 |  |  |  |  |  |  |  |  |  |  |
| IL-1 Signaling | 0.002 | 18 |  |  |  |  |  |  |  |  |  |  |
| IL-8 Signaling | 0.02 | 27 |  |  |  |  |  |  |  |  |  |  |
| Mechanisms of Viral Exit from Host Cells | 0.004 | 10 |  |  |  |  |  |  |  |  |  |  |
| Molecular Mechanisms of Cancer | 0.02 | 44 |  |  |  |  |  |  |  |  |  |  |
| mTOR Signaling | 0.04 | 25 |  |  |  |  |  |  |  |  |  |  |
| Nicotinate and Nicotinamide Metabolism | 0.03 | 16 |  |  |  |  |  |  |  |  |  |  |
| nNOS Signaling in Skeletal Muscle Cells | 0.04 | 4 |  |  |  |  |  |  |  |  |  |  |
| Notch Signaling | 0.0004 | 11 |  |  |  |  |  |  |  |  |  |  |
| Phospholipase C Signaling | 0.004 | 34 |  |  |  |  |  |  |  |  |  |  |
| Protein Kinase A Signaling | 0.04 | 39 |  |  |  |  |  |  |  |  |  |  |
| RAR Activation | 0.006 | 27 |  |  |  |  |  |  |  |  |  |  |
| RhoGDI Signaling | 0.002 | 29 |  |  |  |  |  |  |  |  |  |  |
| Riboflavin Metabolism | 0.04 | 5 |  |  |  |  |  |  |  |  |  |  |
| Sertoli Cell-Sertoli Cell Junction Signaling | 0.01 | 26 |  |  |  |  |  |  |  |  |  |  |
| Signaling by Rho Family GTPases | 0.01 | 33 |  |  |  |  |  |  |  |  |  |  |
| Sonic Hedgehog Signaling | 0.02 | 7 |  |  |  |  |  |  |  |  |  |  |
| Tight Junction Signaling | 0.005 | 25 |  |  |  |  |  |  |  |  |  |  |
| α-Adrenergic Signaling | 0.04 | 14 |  |  |  |  |  |  |  |  |  |  |
|  |  |  |  |  |  |  |  |  |  |  |  |  |
|  |  |  |  |  |  |  |  |  |  |  |  |  |
| **Fibroblasts** | **p-value** | **No of genes** | |  |  |  |  |  |  |  |  |  |
| 14-3-3-mediated Signaling | 0.04 | 13 |  |  |  |  |  |  |  |  |  |  |
| Agrin Interactions at Neuromuscular Junction | 0.01 | 10 |  |  |  |  |  |  |  |  |  |  |
| Aminoacyl-tRNA Biosynthesis | 0.02 | 6 |  |  |  |  |  |  |  |  |  |  |
| Ascorbate and Aldarate Metabolism | 0.03 | 4 |  |  |  |  |  |  |  |  |  |  |
| Axonal Guidance Signaling | 0.04 | 35 |  |  |  |  |  |  |  |  |  |  |
| Bile Acid Biosynthesis | 0.04 | 7 |  |  |  |  |  |  |  |  |  |  |
| CCR3 Signaling in Eosinophils | 0.04 | 13 |  |  |  |  |  |  |  |  |  |  |
| Cdc42 Signaling | 0.04 | 14 |  |  |  |  |  |  |  |  |  |  |
| Chemokine Signaling | 0.04 | 9 |  |  |  |  |  |  |  |  |  |  |
| CXCR4 Signaling | 0.04 | 16 |  |  |  |  |  |  |  |  |  |  |
| EIF2 Signaling | 0.002 | 22 |  |  |  |  |  |  |  |  |  |  |
| Glycine, Serine and Threonine Metabolism | 0.02 | 10 |  |  |  |  |  |  |  |  |  |  |
| Mitochondrial Dysfunction | 0.02 | 15 |  |  |  |  |  |  |  |  |  |  |
| mTOR Signaling | 0.002 | 23 |  |  |  |  |  |  |  |  |  |  |
| Oxidative Phosphorylation | 0.006 | 16 |  |  |  |  |  |  |  |  |  |  |
| Purine Metabolism | 0.01 | 29 |  |  |  |  |  |  |  |  |  |  |
| Regulation of eIF4 and p70S6K Signaling | 0.03 | 16 |  |  |  |  |  |  |  |  |  |  |
| RhoA Signaling | 0.03 | 13 |  |  |  |  |  |  |  |  |  |  |
| SAPK/JNK Signaling | 0.03 | 11 |  |  |  |  |  |  |  |  |  |  |
| Virus Entry via Endocytic Pathways | 0.02 | 12 |  |  |  |  |  |  |  |  |  |  |
|  |  |  |  |  |  |  |  |  |  |  |  |  |
| **iPS-derived neurons (Brennand et al, 2011)** | **p-value** | **No of genes** | |  |  |  |  |  |  |  |  |  |
| Actin Cytoskeleton Signaling | 0.01 | 39 |  |  |  |  |  |  |  |  |  |  |
| Amyotrophic Lateral Sclerosis Signaling | 0.002 | 23 |  |  |  |  |  |  |  |  |  |  |
| Androgen Signaling | 0.003 | 25 |  |  |  |  |  |  |  |  |  |  |
| Aryl Hydrocarbon Receptor Signaling | 0.03 | 25 |  |  |  |  |  |  |  |  |  |  |
| Axonal Guidance Signaling | 0.002 | 70 |  |  |  |  |  |  |  |  |  |  |
| Breast Cancer Regulation by Stathmin1 | 0.03 | 34 |  |  |  |  |  |  |  |  |  |  |
| Cardiac β-adrenergic Signaling | 0.03 | 25 |  |  |  |  |  |  |  |  |  |  |
| Caveolar-mediated Endocytosis Signaling | 0.003 | 18 |  |  |  |  |  |  |  |  |  |  |
| Cellular Effects of Sildenafil (Viagra) | 0.02 | 26 |  |  |  |  |  |  |  |  |  |  |
| Colorectal Cancer Metastasis Signaling | 0.03 | 41 |  |  |  |  |  |  |  |  |  |  |
| CREB Signaling in Neurons | 0.003 | 35 |  |  |  |  |  |  |  |  |  |  |
| CXCR4 Signaling | 0.03 | 28 |  |  |  |  |  |  |  |  |  |  |
| Dopamine-DARPP32 Feedback in cAMP Signaling | 0.009 | 32 |  |  |  |  |  |  |  |  |  |  |
| Ephrin Receptor Signaling | 0.02 | 32 |  |  |  |  |  |  |  |  |  |  |
| G Beta Gamma Signaling | 0.02 | 19 |  |  |  |  |  |  |  |  |  |  |
| G Protein Signaling Mediated by Tubby | 0.03 | 8 |  |  |  |  |  |  |  |  |  |  |
| Germ Cell-Sertoli Cell Junction Signaling | 0.008 | 31 |  |  |  |  |  |  |  |  |  |  |
| Glioma Invasiveness Signaling | 0.005 | 15 |  |  |  |  |  |  |  |  |  |  |
| Glutamate Receptor Signaling | 0.004 | 15 |  |  |  |  |  |  |  |  |  |  |
| Histidine Metabolism | 0.04 | 11 |  |  |  |  |  |  |  |  |  |  |
| Human Embryonic Stem Cell Pluripotency | 0.02 | 25 |  |  |  |  |  |  |  |  |  |  |
| IL-8 Signaling | 0.01 | 35 |  |  |  |  |  |  |  |  |  |  |
| Keratan Sulfate Biosynthesis | 0.003 | 14 |  |  |  |  |  |  |  |  |  |  |
| LPS/IL-1 Mediated Inhibition of RXR Function | 0.03 | 35 |  |  |  |  |  |  |  |  |  |  |
| Molecular Mechanisms of Cancer | 0.04 | 55 |  |  |  |  |  |  |  |  |  |  |
| Neuregulin Signaling | 0.04 | 17 |  |  |  |  |  |  |  |  |  |  |
| Neuropathic Pain Signaling In Dorsal Horn Neurons | 0.006 | 23 |  |  |  |  |  |  |  |  |  |  |
| N-Glycan Biosynthesis | 0.03 | 10 |  |  |  |  |  |  |  |  |  |  |
| Notch Signaling | 0.01 | 10 |  |  |  |  |  |  |  |  |  |  |
| Protein Kinase A Signaling | 0.002 | 57 |  |  |  |  |  |  |  |  |  |  |
| RAR Activation | 0.0001 | 40 |  |  |  |  |  |  |  |  |  |  |
| Retinoic acid Mediated Apoptosis Signaling | 0.002 | 15 |  |  |  |  |  |  |  |  |  |  |
| RhoGDI Signaling | 0.008 | 34 |  |  |  |  |  |  |  |  |  |  |
| Role of NFAT in Cardiac Hypertrophy | 0.02 | 33 |  |  |  |  |  |  |  |  |  |  |
| Role of Wnt/GSK-3β Signaling in the Pathogenesis of Influenza | 0.03 | 15 |  |  |  |  |  |  |  |  |  |  |
| Semaphorin Signaling in Neurons | 0.03 | 12 |  |  |  |  |  |  |  |  |  |  |
| Signaling by Rho Family GTPases | 0.03 | 40 |  |  |  |  |  |  |  |  |  |  |
| Thrombin Signaling | 0.006 | 37 |  |  |  |  |  |  |  |  |  |  |
| Thyroid Cancer Signaling | 0.009 | 11 |  |  |  |  |  |  |  |  |  |  |
| TR/RXR Activation | 0.02 | 18 |  |  |  |  |  |  |  |  |  |  |
| Virus Entry via Endocytic Pathways | 0.02 | 19 |  |  |  |  |  |  |  |  |  |  |
| Wnt/β-catenin Signaling | 0.007 | 33 |  |  |  |  |  |  |  |  |  |  |
| Xenobiotic Metabolism Signaling | 0.02 | 43 |  |  |  |  |  |  |  |  |  |  |
| α-Adrenergic Signaling | 0.03 | 18 |  |  |  |  |  |  |  |  |  |  |
